# Vitality of the Diphtheria Poison

**Published:** 1880-08

**Authors:** 


VITALITY OF THE DIPHTHERIA POISON.—A remarkable proof of the long vitality of the diphtheria poison is communicated from Russia. A boy, a member of an upper class family in South Russia, died of diphtheria four years ago. A new family sepulchre having been just erected it was proposed to inter in it the remains of the child. Before lowering the coffin in the grave, the father of the child had the coffin opened to look at it. The father and all the children, five in number, took a look at the remains through a hole made in the lid of the coffin. On the very next day every one of the children took sick with diphtheria and one of them died of the disease.—*Allgem. Wiener Med. Zeitung, June* 22, 1880.

